# 3D Nanofabrication of SiOC Ceramic Structures

**DOI:** 10.1002/advs.201800937

**Published:** 2018-10-23

**Authors:** Laura Brigo, Johanna Eva Maria Schmidt, Alessandro Gandin, Niccolò Michieli, Paolo Colombo, Giovanna Brusatin

**Affiliations:** ^1^ Department of Industrial Engineering University of Padova Via Marzolo 9 35131 Padova Italy; ^2^ INSTM Padova RU Via Marzolo 9 35131 Padova Italy; ^3^ Department of Physics University of Padova Via Marzolo 8 35131 Padova Italy; ^4^ Department of Materials Science and Engineering The Pennsylvania State University University Park PA 16802 USA

**Keywords:** 3D laser writing, 3D nanofabrication, ceramics, preceramic polymers, two‐photon laser lithography

## Abstract

Shaping ceramic materials at the nanoscale in 3D is a phenomenal engineering challenge, that can offer new opportunities in a number of industrial applications, including metamaterials, nano‐electromechanical systems, photonic crystals, and damage‐tolerant lightweight materials. 3D fabrication of sub‐micrometer ceramic structures can be performed by two‐photon laser writing of a preceramic polymer. However, polymer conversion to a fully ceramic material has proven so far unfeasible, due to lack of suitable precursors, printing complexity, and high shrinkage during ceramic conversion. Here, it is shown that this goal can be achieved through an appropriate engineering of both the material and the printing process, enabling the fabrication of preceramic 3D shapes and their transformation into dense and crack‐free SiOC ceramic components with highly complex, 3D sub‐micrometer architectures. This method allows for the manufacturing of components with any 3D specific geometry with fine details down to 450 nm, rapidly printing structures up to 100 µm in height that can be converted into ceramic objects possessing sub‐micrometer features, offering unprecedented opportunities in different application fields.

## 1 Introduction

Constant advances in the field of 3D manufacturing techniques have pushed resolution limits of these methodologies down to the micro‐ and nanoscale. For example, microscale lattice materials, metamaterials, photonic crystals, nano‐electromechanical (NEMS) and micro‐electromechanical systems (MEMS) are taking advantage of sub‐micrometer‐scale feature size effects in 3D.^1^, ^2^, ^3^, ^4^, ^5^, ^6^, ^7^, ^8^ In these contexts, fabrication of components based on ceramic materials would expand the range of properties offered by polymers currently used for 3D nanoscale fabrications, allowing for higher refractoriness, increased chemical durability, better wear and oxidation resistance, higher elastic modulus and improved dimensional stability with temperature.^9^, ^10^, ^11^, ^12^, ^13^

However, shaping preceramic polymers at the sub‐micrometer scale while generating complex 3D ceramics architecture has been so far unfeasible: scarce examples reported in literature present limitations of poor definition of 3D forms and incomplete ceramization,^14^, ^15^, ^16^, ^21^, ^22^, ^23^, ^24^ due to lack of suitable precursors, high shrinkage accompanying the heat treatment, and pyrolysis temperature below the completion of the polymer‐to‐ceramic transformation (heating below 800 °C). Indeed, methods for 3D printing at high resolution typically employ two‐photon lithography (2PL), triggering polymerization of a photosensitive resists.^17^, ^18^, ^19^ Differently from photosensitive organic resins, preceramic polymers (precursors for SiOC, SiCN, or SiC materials) are a unique class of polymers that combine the processability properties of polymeric materials with the capability of transforming into ceramics through high‐temperature treatments in inert or oxidative atmospheres.^20^

Overcoming current limitations, we have here engineered an unprecedented fabrication procedure able to generate sub‐micrometer 3D complex architectures (with size up to 100 µm) of stable, dense (pore‐free), crack‐free, and oxidation resistant SiOC ceramic compositions. These nanostructures are fully ceramized without shape distortion during the pyrolysis step, thanks to a synergistic combination of the functional preceramic polymer, a proper initiator and a novel engineered configuration for the fabrication of the 3D structures. This work represents the first example of the straightforward, fast, and versatile 3D fabrication of fully ceramic SiOC sub‐micrometer‐sized 3D, components with dimensions of several tens of micrometer and minimum feature size down to about 450 nm. The availability of ceramic sub‐micrometer shapes with these characteristics will open up a host of opportunities in different fields, where their superior properties allow ceramics to outperform current materials. Just to give an example, microneedle arrays for transdermal delivery of pharmacologic agents manufactured using biologically compatible ceramics possess higher mechanical strength and better stability at high temperature and humidity than most polymers, and allow for high performance insertion into the skin.^25^, ^26^ Rather than using polymeric materials, it would also be advantageous to manufacture micronozzles with SiOC ceramics possessing better stiffness, superior wear and chemical resistance and high dimensional stability with varying temperature, for applications where precisely mixing or dispensing gases or liquids is required. Other examples include components for microfluidics and NEMS or MEMS operating in harsh environments.

## 2 Fabrication and Pyrolysis of Structures

**Figure 1:**
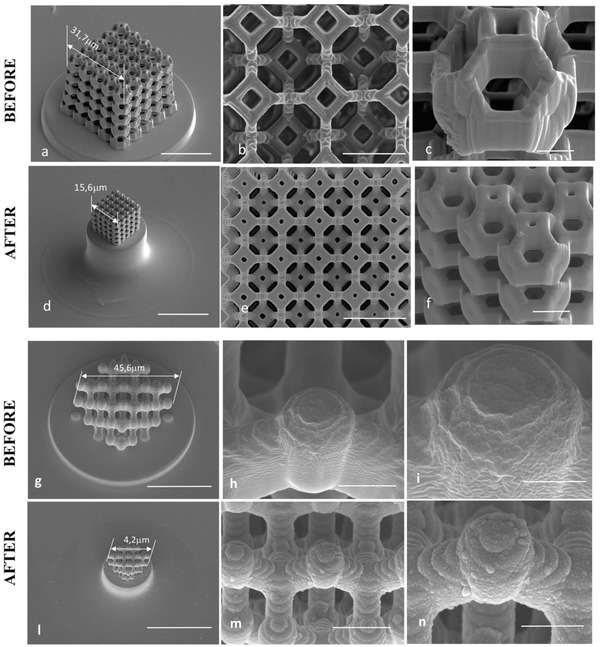
SEM images of a a–f) KC and a g–n) DS, fabricated at laser power 50% and scan speed 2000 µm s^−1^, a–c,g–i) before and d–f,l–n) after pyrolysis at 1000 °C, at different magnifications. All SEM before and after pyrolysis were acquired with the same magnification factor. A linear isotropic shrinkage of 51% (KC) and 56% (DS), with respect to the printed structures, was measured. Scale bars: 30 µm (a,d), 5 µm (b,e), 3 µm (c,f), 30 µm (g,l), 5 µm (h,m), 3 µm (i,n).

**Figure 2:**
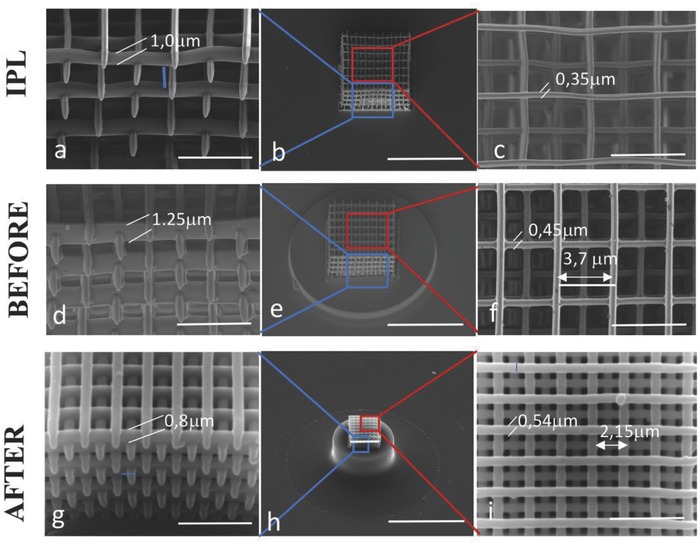
a–c) Fabrication of woodpiles with scan speed of 450 µm s^−1^, power scaling 1, laser power 50, using IPL (nanoscribe proprietary resist). d–f) The line dimension is approximately an oval section, 350 nm wide and 1 µm tall. Fabrication of woodpiles with the same scan speed and power scaling, laser power 70 and *z*‐scaling 1.5, using the preceramic polymer. The line dimension is approximately an oval section, 0.45 µm wide and 1.25 µm tall. g–i) Pyrolyzed preceramic woodpile with line dimension 0.54 µm in width and 0.8 µm in height, indicating a linear shrinkage of 42% and 36% along the pile and in the *z*‐directions respectively, producing a thickening in the lateral dimension of the pile. The overall shrinkage of the woodpile is 54%. Scale bars: 40 µm (b,e,h), 5 µm (a,c,d,f,g,i).

We selected a few significant 3D shapes to validate the feasibility of shaping preceramic polymers at the sub‐micrometer scale: the Kelvin cell (KC, **Figure** **1**a–f) and the Diamond structure (DS, Figure 1g–n) represent examples of complex, porous, and highly detailed architectures which validate the possibility of generating long overhangs and demonstrate the freedom of 3D design that this technology affords, while woodpile structures were chosen because their simple shape allows to test the intrinsic limitation of the process in terms of the smallest feature dimensions achievable (**Figure** **2**).

KC and DS were fabricated after optimization of process parameters (example in Figure S1 in the Supporting Information) and converted to SiOC crack‐free ceramic microsized 3D components. After pyrolysis at 1000 °C (Figure 1), no significant shape distortion in the 3D was observed. Remarkably, this was achieved in spite of the overall linear shrinkages of 51% (KC) and 56% (DS), with respect to the printed structures (Figure 1) that, in terms of volume shrinkage, resulted in a contraction of almost 90%.

The lack of evident deformation of the overall architecture or melting during pyrolysis indicated that no softening or bloating occurred during ceramization: this indicates that a suitably high degree of cross‐linking was achieved during the two‐photon polymerization. This is an important and key issue when 2PL is used, from which not only the shape preservation during thermal treatment depends but also the prevention of collapse during the development step, i.e., the chemical treatment used to remove uncrosslinked polymer (Figure S1 on the far left in the Supporting Information). Likewise, a sufficient degree of crosslinking of the polymer should be achieved in a reasonable period, as fabrication time is a decisive parameter to develop a promising process technology. To this end, we conducted this work deliberately disregarding material formulations showing low throughput compared to standard two‐photon resists.

## 3 Preceramic Formulation and Printing Configuration

**Scheme 1:**
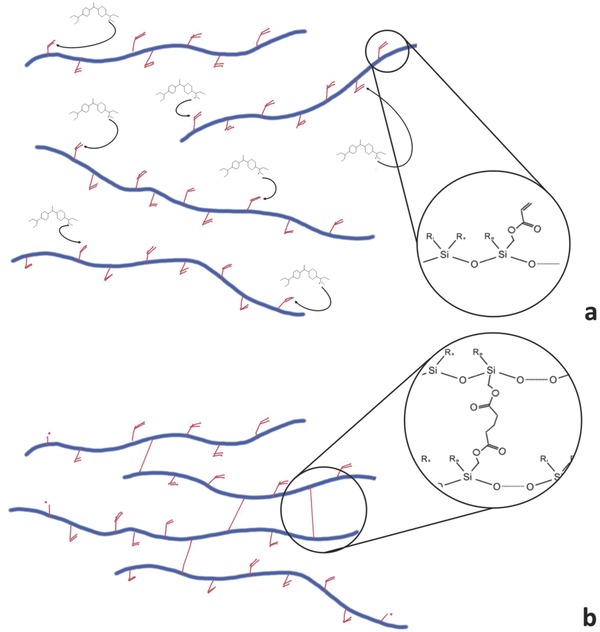
a) BDEBP radical activation by 2 photon absorption and lateral acrylate initiation, b) crosslinking by polymerization of the siloxane preceramic polymer.

Our preceramic formulation is shown in **Scheme** **1**a and was based on a commercial, solvent‐free acrylate siloxane, RC 711, and a Michler's ketone derivative molecule, bis(dimethylamino)benzophenone (BDEBP), generally used as one‐photon radical polymerization initiator at about 365 nm (Figure S2, Supporting Information) but known to be able to efficiently activate radical polymerization by two‐photon absorption. Thanks to the donor–acceptor–donor structure, upon two‐photon absorption at 780 nm, the carbonyl group of BDEBP is promoted to an excited singlet state, that can undergo an intersystem crossing to the triplet state, from which two radical ions are produced via electron donation by the amine to the carbonyl moieties.^27^ During direct laser writing, a femtosecond pulsed laser is tightly focused into a small volume of the photosensitive preceramic formulation, a voxel, whose ellipsoid shape dimensions range from less than 1 µm (in width) to a few µm (in height). Within this volume, two‐photon polymerization rapidly occurred, Scheme 1b, thanks to the high efficiency of the initiator BDEBP, selected for its high dispersion in the siloxane resin RC 711, and the high amount of acrylated reactive groups provided by the siloxane. This combination was capable of forming a continuous crosslinked polymer network by radical polymerization (Scheme 1b), at the same time allowing for a high fabrication speed, up to 50 000 µm s^−1^. This performance is close to the highest achievable with standard resists (60 000 µm s^−1^) and very uncommon for nonstandard two‐photon laser writing resists, as demonstrated for the fabrication of simple structures (Figure S3, Supporting Information).

**Scheme 2:**
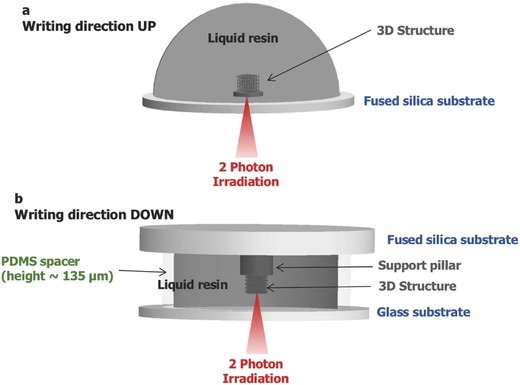
Schematics of the “standard” approach ( a) writing direction: UP) and “inverted” configuration ( b) writing direction: DOWN) for 3D fabrication. a: The standard approach is an additive process in which the laser beam is initially focused at the lower glass/resin interface, and successively moved layer‐by‐layer at increasing *z*‐coordinates, according to the 3D design, for polymerizing the whole structure. Power compensation is required for assuring a constant exposure dose at increasingly higher penetration depths within the liquid and through just‐polymerized layers. Due to the working distance of the objective, a maximum height of the fabricated structures of ≈100 µm on a thin glass slide (150 µm) is possible. b: Alternative “inverted” approach developed to fabricate higher structures on thick (1 mm) fused silica substrate, due to the requirement to have a support pillar on the substrate for subsequent pyrolysis. A preceramic polymer solution drop is placed between two glass substrates, separated by a thin polydimethylsiloxane (PDMS) membrane serving as gasket. The laser beam is initially focused at the upper glass/resin interface, and fabrication proceeds in a layer‐by‐layer fashion for decreasing *z*‐coordinates, eliminating shadowing effects from previously polymerized layers.

**Figure 3:**
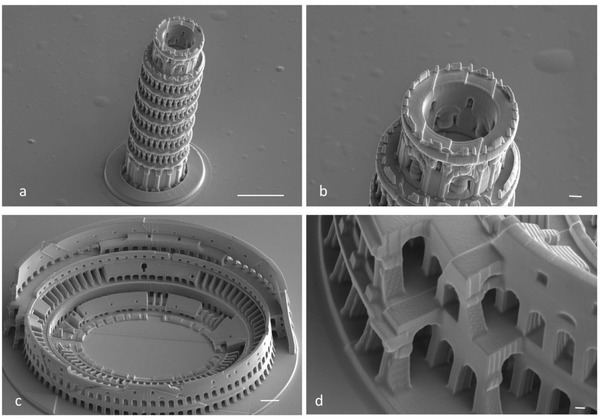
Examples of additional tall and complex 3D structures produced using the preceramic resist (not pyrolyzed): a,b) Pisa tower fabricated at a power scaling of 1.0, laser power 50% and scan speed of 3000 µm s^−1^. c,d) Colosseum, fabricated at a power scaling 1.2, laser power 50%, and scan speed of 2000 µm s^−1^. Scale bars: 20 µm (a,c), 2 µm (b,d).

Once we achieved this, we then proceeded investigating how to maximize fabrication performance; in particular, we searched for the largest dimensions that could be fabricated with a complex shape, compatible with the preservation of voxel size and fabrication speed at all fabrication depths. In fact, when using 2PL frequently encountered drawbacks are the focus distortion and laser power loss, due to shadowing effects when the laser passes through already solidified volumes to move the focus at increasing *z*‐coordinates. This is especially common when nonstandard resist materials are used, as in our case. The success of our approach was achieved by engineering a nonstandard printing configuration (**Scheme** **2**). With this setup, we could grow structures on supports of different thickness, depending on the dimension and geometry of their shape, which were prefabricated on glass (fused silica) substrates thus eliminating the need for power compensation when writing at increasing depths (as it occurs in the standard configuration, Scheme 2). In fact, this approach enabled to avoid shadowing effects, and assured a constant exposure dose at increasingly higher penetration depths within the liquid and through just‐polymerized layers. Because of this, it was possible to maintain a constant fabrication resolution from the bottom to the top of the 3D structure, as the focus was not going through the polymerized layers. **Figure** **3** shows examples of the successful fabrication of complex structures using the configuration reported in Scheme 2, with a height up to 0.1 mm, which would have otherwise been limited to an about ten times smaller dimension, if the standard fabrication configuration was used.

**Figure 4:**
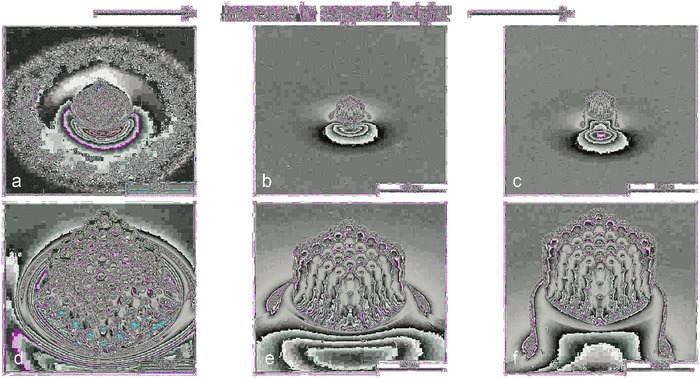
a–c) Pyrolyzed Kelvin cell structure (scaling 1.8 and pyrolyzed at 1000 °C) on support pillars with increasing height, to reduce shrinkage constraints from the glass substrate during pyrolysis. d–f) Figures in the bottom row represent a magnification of the samples shown in the upper row. Scale bars: 40 µm (a–c), 10 µm (d–f).

This result was of critical importance for the final success of our 3D ceramic fabrication approach, and prompted us to implement this nonstandard printing configuration to grow preceramic structures on cylindrical supports. They were specifically designed to reduce constraints from the glass substrate during the shrinkage accompanying the siloxane decomposition. The importance of the presence of a suitable support is shown in **Figure** **4**, where deformation of the 3D structures was observed after pyrolysis when they were grown on support pillars of insufficient height. It is evident how, in the standard fabrication configuration in which the maximum height would be limited to about 10 µm, structures like these could maintain their shape after pyrolysis only by reducing the shrinkage, as when incomplete ceramization is carried out (e.g., by pyrolysis at 600 °C), or by filling the preceramic formulation with powders, to the detriment of the sub‐micrometer finest details and the polymerization efficiency, because of light scattering.^16^ In contrast, we demonstrated in this work that it is possible to obtain fully ceramized final shapes with height of a few micrometer, sacrificing part of the size of the building envelope in the *z*‐direction because of the need to have supports with the proper height but, at the same time, dramatically increasing the fabrication depth by using the newly developed, nonstandard printing configuration. We believe that, in future experiments, substrates consisting entirely of the same preceramic material could be used, thereby avoiding any shape deformation during pyrolysis and leading also to the possibility of integrating structures at different dimensional scales.

**Figure 5:**
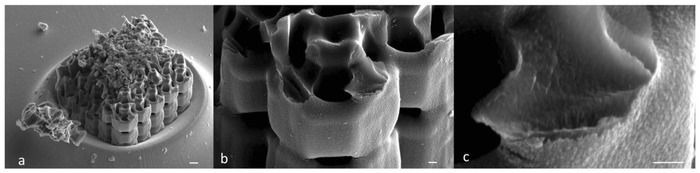
SEM image of a a) broken Kelvin cell structure and b,c) its magnifications; sample pyrolyzed at 1000 °C. From the magnification (c), the presence of a fully dense, crack‐free ceramic material is confirmed. Scale bars: 1 µm (a), 200 nm (b,c).

It is noteworthy that our ceramic structures, generated after pyrolysis at 1000 °C, are pore‐ and crack‐free with a density of 1.98 ± 0.02 g cm^−3^, despite a mass loss larger than 90% (**Figure** **5**). The SiOC ceramic is X‐ray amorphous (Figure S5, Supporting Information).

## 4 Complexity and Resolution of Ceramic Fabrication

We next proceeded to investigate what is the smallest feature dimension achievable by this fabrication method. In order to assess this, we fabricated the highly complex KC and DS 3D shapes shown in Figure 1, but scaled their dimension by a factor between 2 and 3 (Figure S6, Supporting Information), and investigated the smallest features dimensions that could be produced. Remarkably, shape complexity and fine features were maintained at all scaling factors, resolving features with size down to about 0.8 µm. However, the writing parameters used to design these structures impose that the voxel is scanned multiple times across the features until full polymerization is achieved, according with its dimension. Therefore, to stretch the upper limit of this technology applied to our preceramic formulation, we designed simpler 3D structures, such as woodpiles, printed by moving the laser focus along each pile with a single scan. In this fabrication, single line section and 3D size were determined by the voxel shape that was, in turn, affected by the photosensitive material used and the writing parameters and settings. As shown in Figure 3e–g, a single pile fabricated after optimization of the process parameters (Figure S7, Supporting Information) possessed an approximately ellipsoidal section with axes of 0.6 and 2.2 µm in the preceramic material (Figure 3e1, 3) and smaller, about 0.55 and 1.5 µm, for the standard Nanoscribe resist IPL, with larger vertical axes, as expected. These single line sections were therefore an indication of the ultimate feature dimension that can be written with these two materials, relatively to this geometrical shape. Clearly, optical properties of the preceramic polymer, such as refractive index not optimized for 2PL as in the standard resist, provoked voxel deformation (defocussing) that enlarge the minimum pile section size achievable with the preceramic. After pyrolysis (Figure 3g1,3), shrinkage in the *z*‐axis of the pile section was about 64%, giving a final dimension of 800 nm, whereas the pile width remained almost undeformed. This considerable difference can be justified by a different crosslinking degree of the polymer along the vertical direction of the pile section, due to the weaker light intensity of the voxel along this path upon defocussing.

It is noteworthy to observe that the sizes of the features in the component, after pyrolysis, were smaller than those in the as printed state, therefore providing resolutions beyond what the characteristics of the photosensitive polymer employed in the fabrication allow, and approaching those achieved with standard photo‐resists.

## 5 Conclusions

In conclusion, in this work we showed that, using 2PL with an engineered printing configuration and starting from a selected preceramic formulation, we could fabricate 3D complex architectures with size of the order of 0.10 mm in the *z*‐direction, with details down to 450 nm. The achieved structures, after pyrolysis, were dense and fully ceramized into SiOC, overcoming limitation of previously reported examples^10^ and allowing to fully exploit the mechanical, thermal, and chemical resistance typical of ceramics. Besides, our nonstandard printing configuration allowed for printing up to the maximum thickness permitted by this optical set‐up, preserving sub‐micrometer feature details and therefore broadening possible exploitation fields in different technological applications. In this respect, it is noteworthy to point out that this resist formulation and fabrication approach allowed for printing speeds comparable with those of standard photoresists.

## 6 Experimental Section

*Two‐Photon Sensitive Resin*: The liquid photosensitive preceramic resin used in this work was a commercial acrylate siloxane of proprietary composition (TEGO RC 711, Evonik Industries, Germany), with the addition of 1 wt% 4,4′‐bis(diethylamino)benzophenone (BDEBP) radical initiator (Scheme). From the datasheet, the resin density is ≈1 g cm^−^^3^, and it has a viscosity of ≈600 mPa at room temperature. Magnetic stirring at 60 °C overnight was performed to guarantee a complete solubilization of the initiator in the siloxane polymer. The lack of added solvent guarantees high stability of the solution during laser writing, avoiding possible evaporation, and phase separation. The one‐photon absorption spectra for the initiator are reported in Figure S2 in the Supporting Information.

*Two Photon Laser Lithography*: Two‐photon lithography was carried out using a Nanoscribe GT Photonic Professional device. The system was equipped with an erbium‐doped femtosecond laser source, with a center wavelength of 780 nm and power of about 150 mW at a pulse length between 100 and 200 fs. A 63 × 1.4 NA oil immersion objective of 360 µm working distance was used. Nanoscribe GT was calibrated to about 50 mW power in the sample plane, for a power scaling value of 1.0 (returned by the acousto‐optic modulator calibration) and 100% laser power. An estimate of the effective power during a fabrication process was provided by multiplying 50 mW by laser power and power scaling values. Laser power determines the intensity within the focal spot, and therefore exposure dose can be increased either by increasing the laser power or by decreasing scan speed, thus increasing exposure time. Such dose is roughly proportional to the square root of the scan speed value. The laser beam was focused through a glass substrate (a coverslip) into the photosensitive polymer solution; in this configuration, the maximum structure height is limited by the objective working distance and by the substrate thickness (equal to about 150 µm).

Two different configurations were adopted for the fabrication of the structures (Scheme 2). Considering the inverted microscope configuration of Nanoscribe GT, the standard approach (UP, Scheme 2) consists in an additive process in which the laser beam is initially focused at the lower glass/resin interface, and successively moved layer‐by‐layer at increasing *z*‐coordinates, according to the 3D design, for polymerizing the whole structure. In practice, power compensation is required for assuring a constant exposure dose at increasingly higher penetration depths within the liquid and through just‐polymerized layers. Moreover, the fabrication of structures onto millimeter‐thick, fused silica slides is forbidden by the working distance of the objective.

Thus, an alternative “inverted” approach (DOWN) was developed to fabricate the structures needing a support pillar for subsequent pyrolysis on a fused silica glass substrate (Scheme 2). A preceramic polymer solution drop was placed between two glass substrates, separated by thin PDMS membrane serving as gasket. The upper fused silica glass slide (arbitrarily thick) served as support for the growth of the 3D structures, while the lower coverslip separated the objective submerged in oil from the preceramic resin. In this case, the laser beam was initially focused at the upper glass/resin interface, and fabrication proceeded in a layer‐by‐layer fashion for decreasing *z*‐coordinates, eliminating shadowing effects from previously polymerized layers. The fused silica glass slide can withstand heating temperatures in excess of 1000 °C, and was therefore a suitable substrate for pyrolysis of the printed preceramic structures.

All substrates, onto which polysiloxane structures were fabricated, required a previous functionalization with a (3‐methacryloxypropyl)trimethoxysilane solution, in order to provide surface methacrylate groups for covalent binding of the acrylate siloxane to the glass surface. This step guaranteed stable linkage between the 3D solid and the glass substrate during development and during pyrolysis, which was necessary for handling fabricated micro‐ and nanoobjects.

Arbitrarily complex 3D structures were fabricated according to computer‐aided design (CAD) models, providing STL files to the control software for laser writing. For resolution tests, woodpile structures were fabricated. Woodpile structures were drawn in hand‐written Nanoscribe's General Writing Language code as trajectories to be followed by the laser focus inside the polymer solution. A series of woodpile structures of 40 × 40 µm^2^ base area, with lateral rod spacing of 5 µm and axial rod spacing in the range of 0.50–1.25 µm, were written by tuning laser power values (from 10% to 100%) at a fixed scan speed of 450 µm s^−1^, for a power scaling of 1.0 (corresponding to 50 mW laser power on the sample). Structures which were tens of micrometers in height were produced as successive stacks of the basic four‐layer module of a woodpile.

*Pyrolysis of the Preceramic Structures*: After 3D laser writing, removal of the unpolymerized solution was performed with diphenyl ether using a pipette to promote solvent flow on the glass substrate and the just fabricated structure. Once cleaned, samples were dried at 60 °C on a heating plate for at least 1 h to remove any trace of cleaning solvent from the surface. Samples were then pyrolyzed in an alumina tube furnace (Lindberg, Riverside, MI) at 800 and 1000 °C for 1h in nitrogen (99.99%) with a heating rate of 2 °C min^−1^.

*Characterizations*: Thermogravimetric analysis of the UV‐cured preceramic polymer (TGA, STA 409/429 Netzsch, Verona, Italy) was carried out at a heating rate of 5 °C min^−1^ in 99.99% flowing nitrogen.

The optical absorbance of the preceramic polymer and of the photoinitiator (dissolved in toluene at a 0.1 wt% concentration) was investigated by UV–vis spectrometry (V‐650, JASCO International Co., Japan).

XRD spectra were collected with a Bruker AXS D8 diffractometer, with Cu‐Kα radiation and θ–2θ configuration. Characterized samples were powders produced from finely milled UV‐polymerized samples after pyrolysis at 800, 1000, and 1200 °C. The same samples were also characterized with a helium pycnometer (AccuPyc 1330. Micromeritics, Milan, Italy) to provide an estimate of the SiOC density as a function of the pyrolysis temperature.

High resolution imaging for the 3D structures was conducted by scanning electron microscopy (SEM, Quanta 450, FEI, USA) on Au‐coated samples.

## Conflict of Interest

The authors declare no conflict of interest.

## Supporting information

Supplementary
