# Supplementary material for: 3D Nanofabrication of SiOC Ceramic Structures
Source: Adv Sci (Weinh). 2018 Oct 23;5(12):1800937. doi: 10.1002/advs.201800937 (PMC6299732; doi:10.1002/advs.201800937)
Supplement: Supplementary file 1 — Supplementary [file ADVS-5-1800937-s001.pdf]

Copyright WILEY-VCH Verlag GmbH & Co. KGaA, 69469 Weinheim, Germany, 2018.

ADVANCED SCIENCE
Open Access

Supporting Information

for *Adv. Sci.,* DOI: 10.1002/advs.201800937

3D Nanofabrication of SiOC Ceramic Structures

*Laura Brigo, Johanna Eva Maria Schmidt, Alessandro Gandin, Niccolò Michieli, Paolo Colombo, and Giovanna Brusatin**

SUPPLEMENTARY INFORMATION

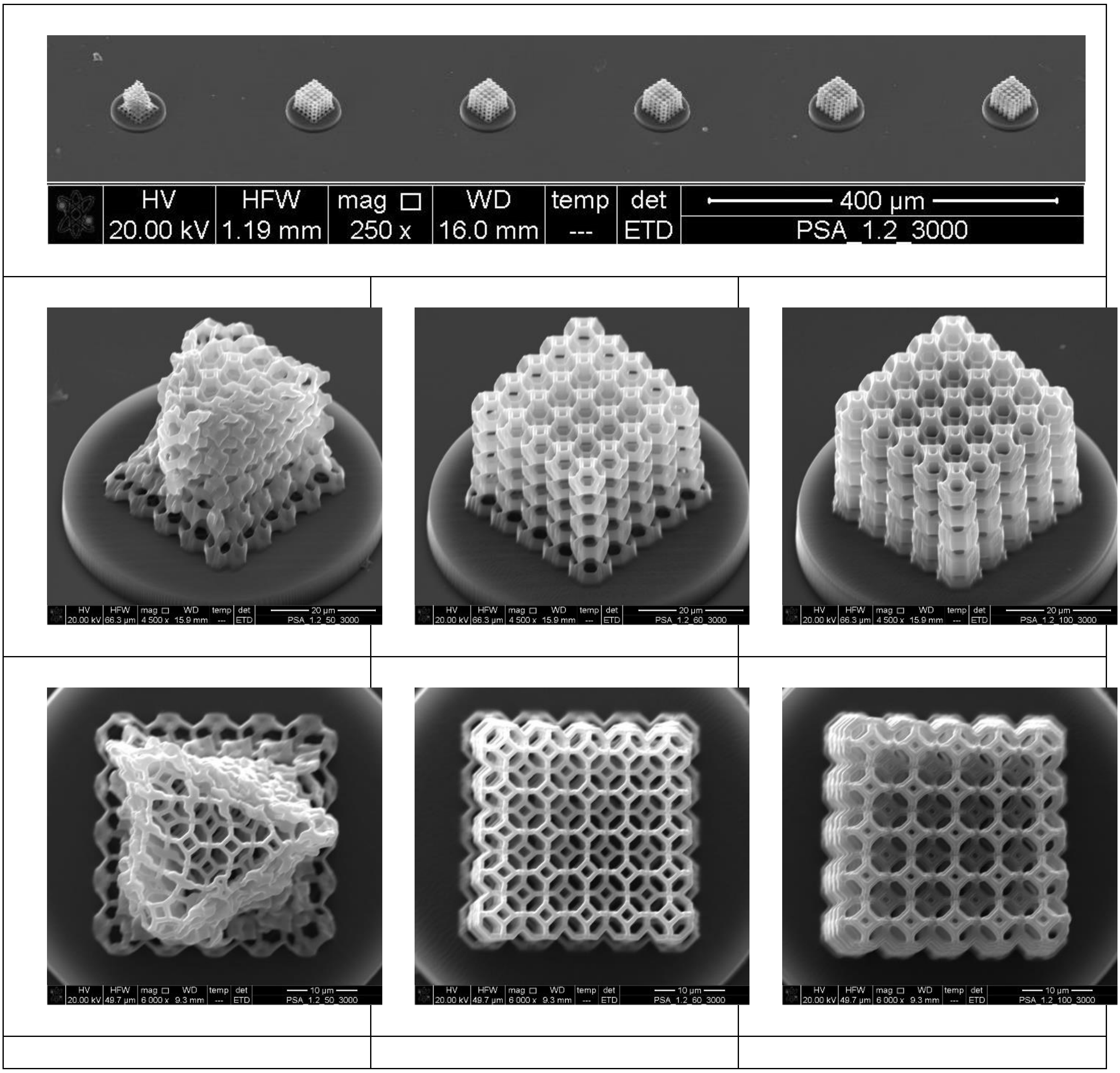

Figure S1. optimization of process parameters made through dose matrices from underexposed (far left) to overexposed (far right) fabrications with the preceramic polymer. Fabrication parameters were changed exploring a range of laser power, power scaling and scan speed values, therefore tailoring the final exposure dose.

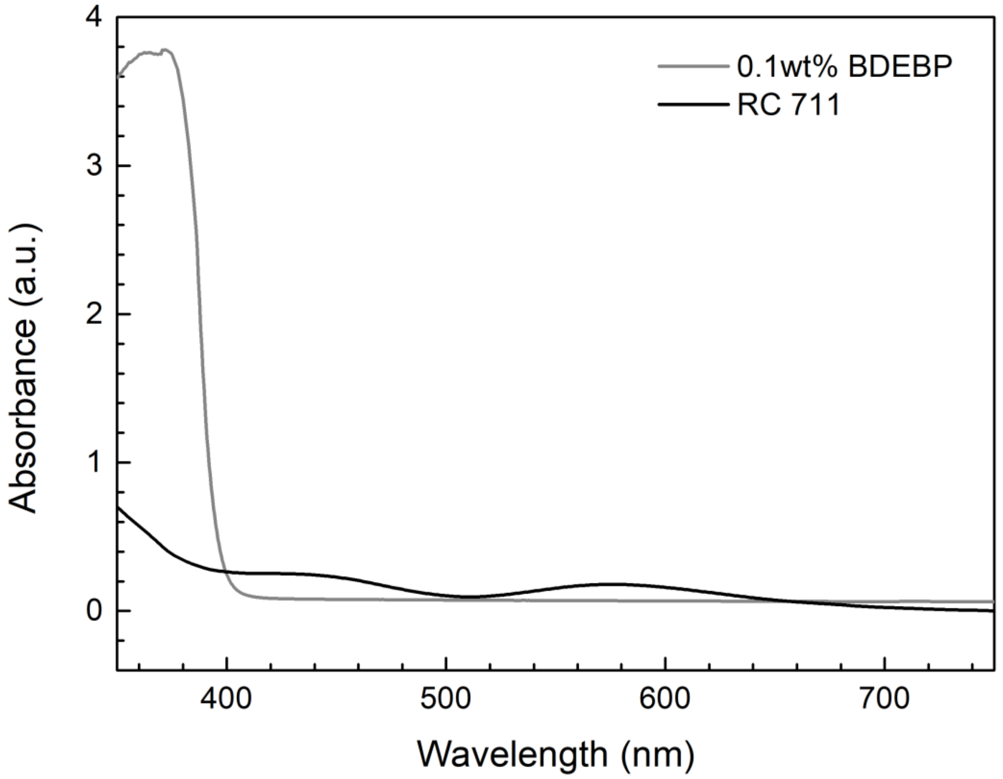

Figure S2. Single photon UV-vis absorbance of the RC 711 preceramic polymer and of the used BDEBP initiator (dissolved in toluene at a 0.1 wt% concentration).

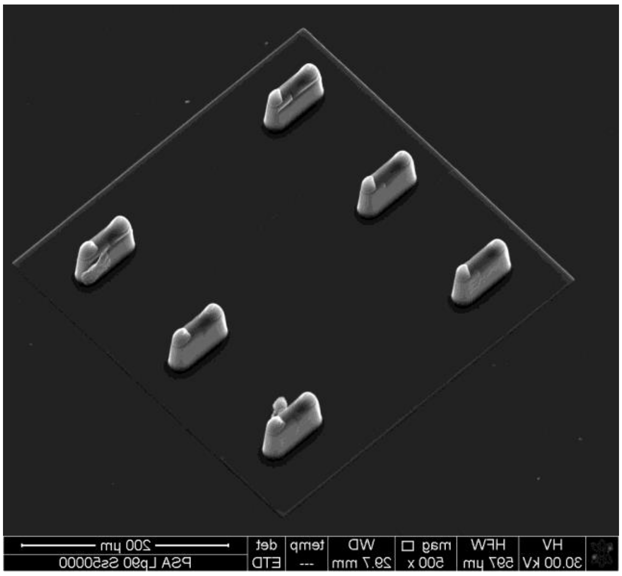

Figure S3. Simple pillars, fabricated with the preceramic polymer with scan speeds up to 50000 μm/s.

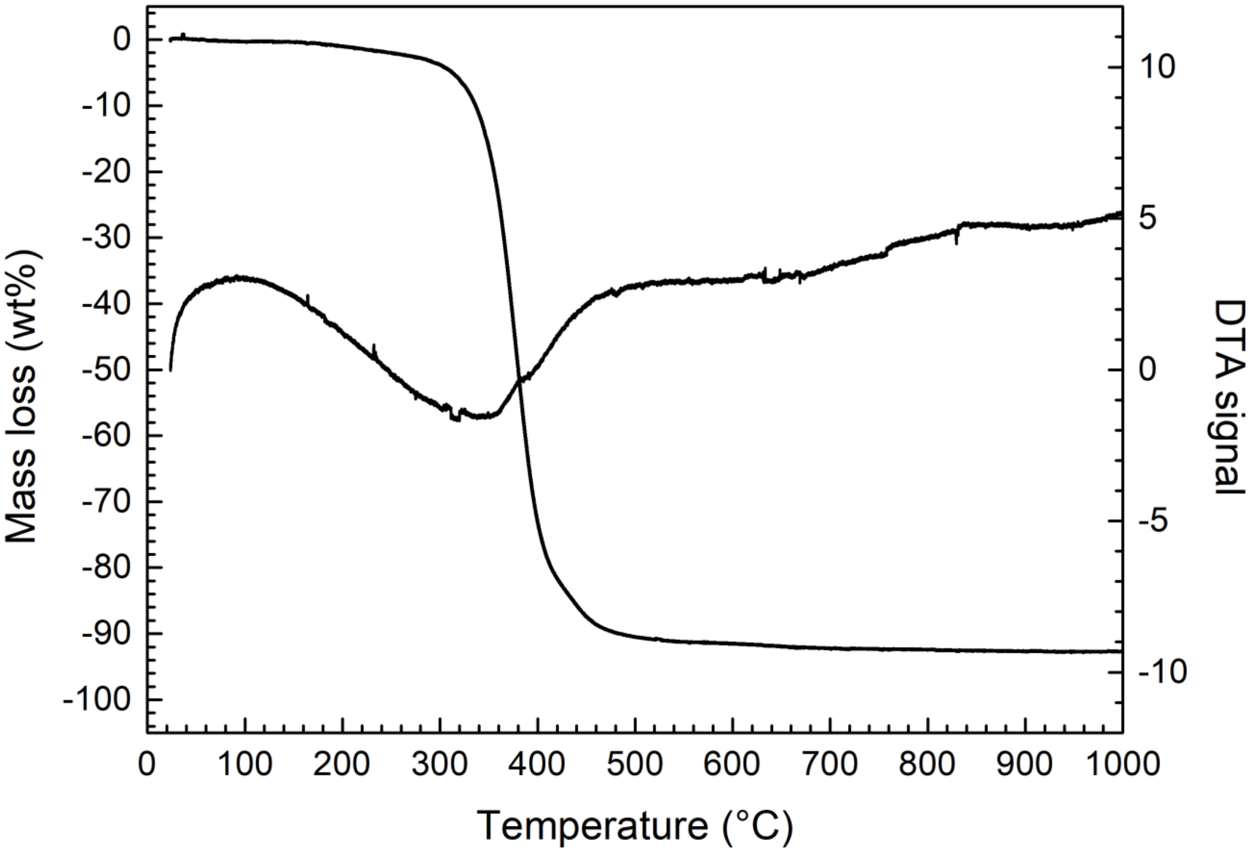

Figure S4. TG/DTA analysis of mass change and temperature difference, reported as a function of the temperature, for RC 711. Significant weight loss takes place in the temperature range of 350–500°C, due to the pyrolysis of the preceramic polymer, giving a ceramic yield of 7.4 wt% at 1000°C.

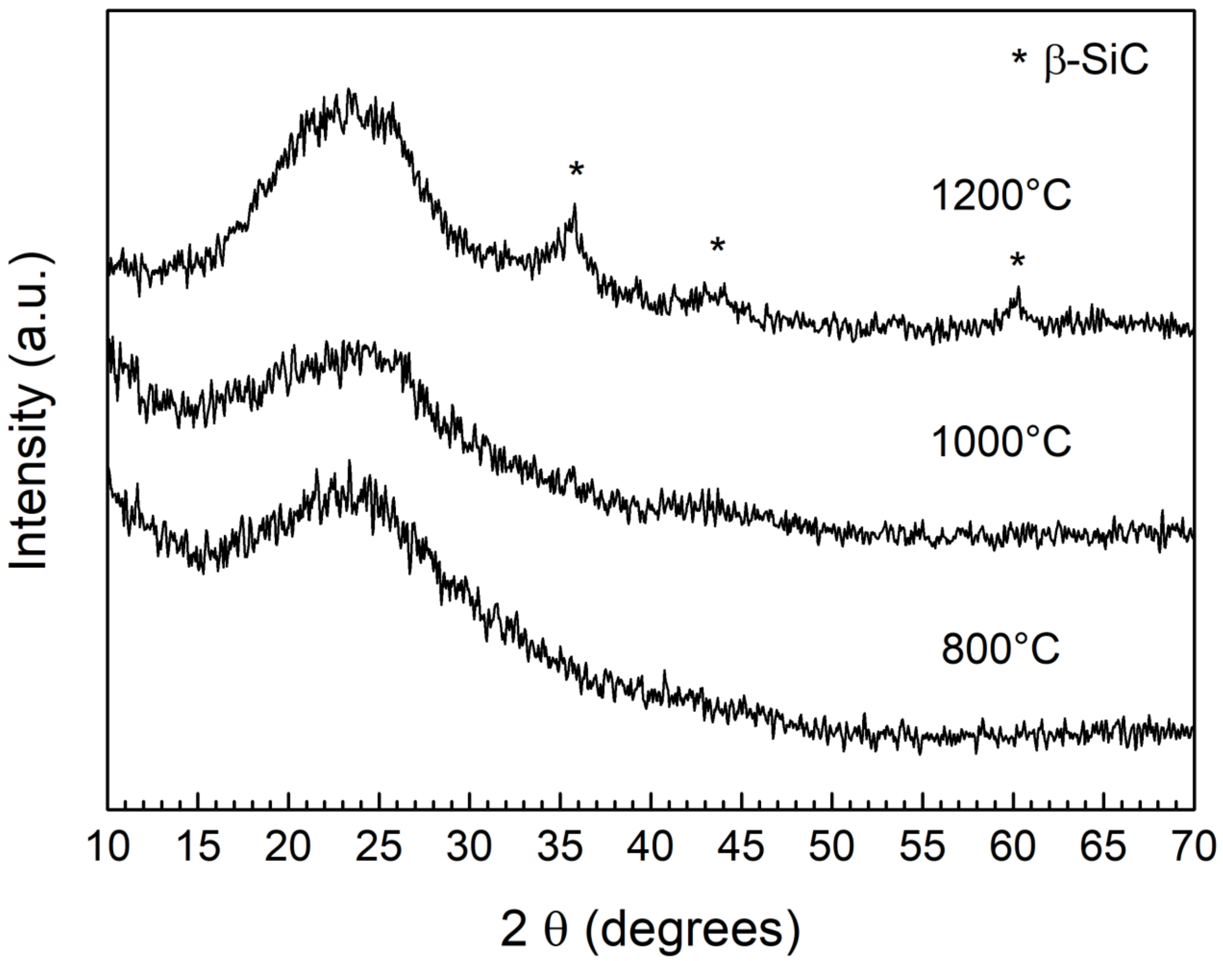

Figure S5. XRD patters of the preceramic polymer pyrolyzed at different temperatures. The material remains amorphous upon treatment at 800 and 1000°C, and contains some β-SiC nano-sized crystals after treatment at 1200°C.

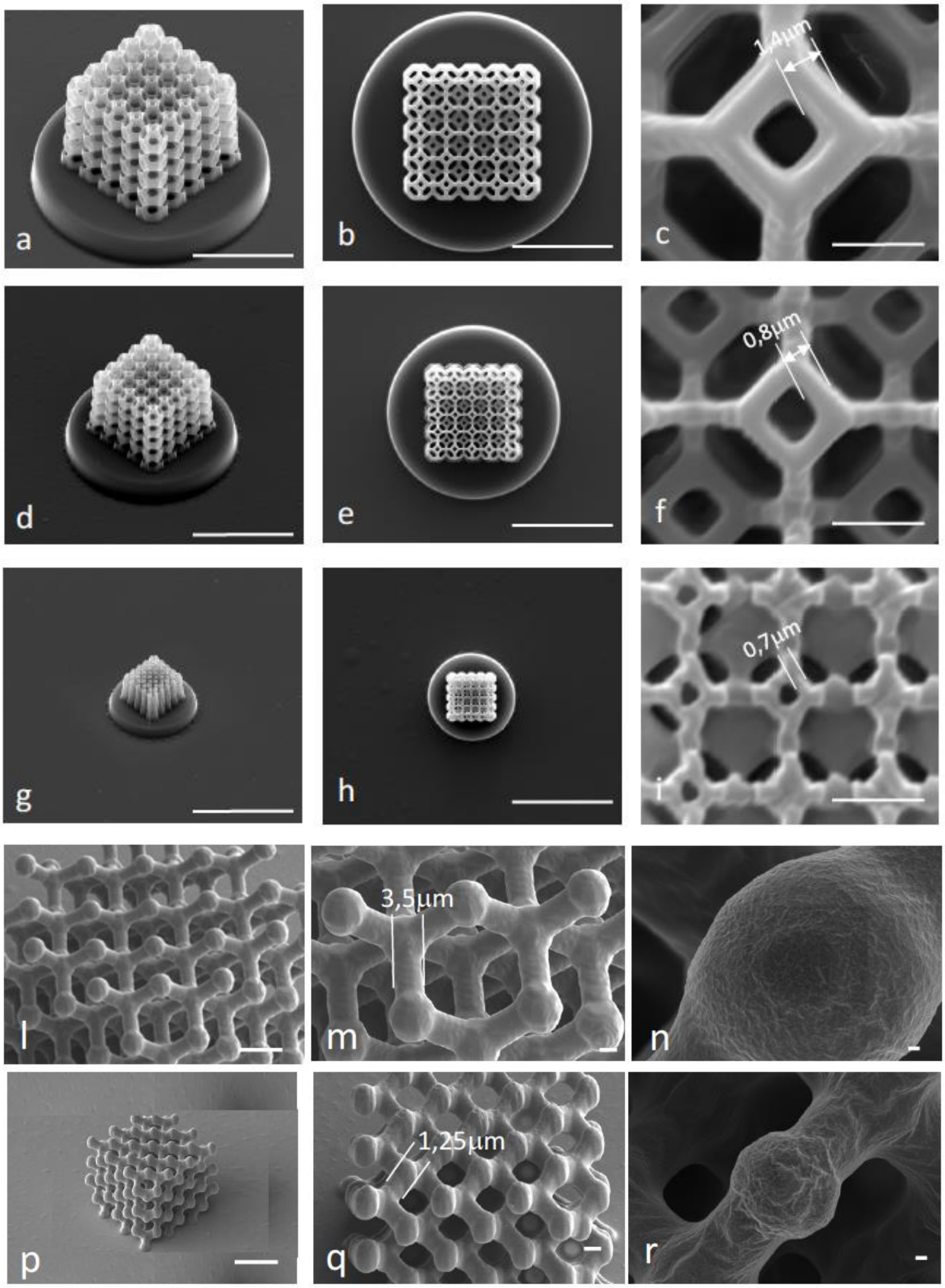

Figure S6. 3D fabrication of preceramic structures with different and complex architectures, before pyrolysis. SEM images of Kelvin cell structures (a-i) fabricated after optimization of the process parameters (example in Figure S1) at a power scaling 1.2, laser power between 70 and 90% and scan speed of 2000 µm/s with different scaling factors (a,b,c: 2.75, d,e,f: 2.0, g,h,i: 1.0). Measured struts dimensions are: 1.4, 0.8 and 0.7 µm for the three scaling factors respectively, with scaling 2 presenting a slightly thinner strut, probably due to the lower laser power used for the fabrication, while struts in scaling 1 are 60% larger. The correct scaling of the distance from the

middle of two struts in the small rectangle was also maintained, scaling from 3.3 to 2.2 and 1.1 µm (distance measured from the center of two struts).
SEM images of Diamond structures (l-r) fabricated after optimization of the process parameters. Two different scaling factors (l-n: 3, p-r: 1). Rods connecting diamond nodes have diameters of 3.5 and 1.25 µm for the two scaling factors respectively, indicating a diameter dimension slightly larger with scaling factor 1. The correct scaling of the distance from the middle of two rods was almost maintained, scaling from 15 to 5.5 µm (m and q, distance measured from the center of two rods).
Scale bars: 40 µm (a,b,d,e,g,h), 3 µm (c,f,i), 10 µm (l,p), 2 µm (m,q), 200 nm (n,r).

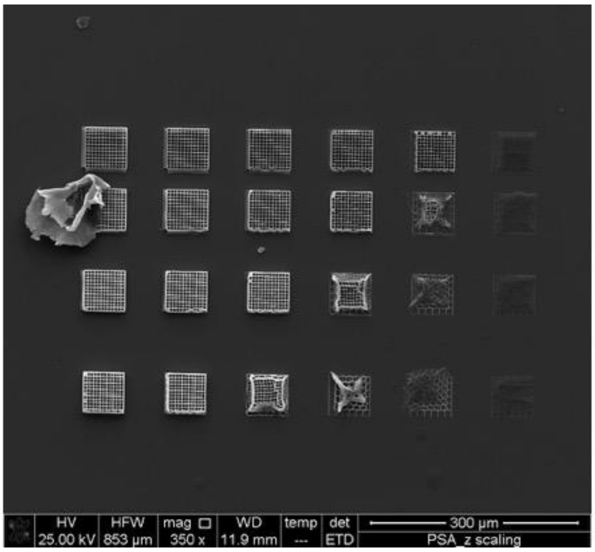

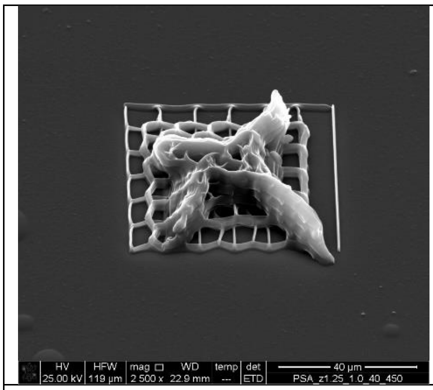	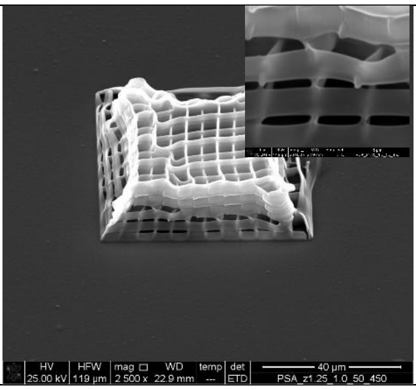	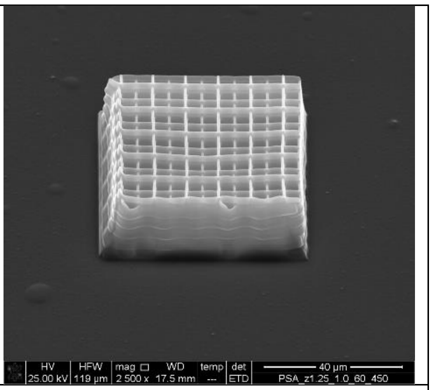
Not enough laser power → collapse	Border → maximum z-resolution	Completely standing, but already closed side holes

Figure S7. Dose matrix (a) and some magnifications (b,c,d) of woodpiles fabricated with the preceramic polymer with a distance between piles of 1.5 µm (Figure 3) and placed over a layer of identical piles oriented orthogonally and with a variable distance in the z direction.
